# Reversing Interfacial Catalysis of Ambipolar WSe_2_ Single Crystal

**DOI:** 10.1002/advs.201901382

**Published:** 2019-12-05

**Authors:** Zegao Wang, Hong‐Hui Wu, Qiang Li, Flemming Besenbacher, Yanrong Li, Xiao Cheng Zeng, Mingdong Dong

**Affiliations:** ^1^ College of Materials Science and Engineering Sichuan University Chengdu 610065 China; ^2^ Interdisciplinary Nanoscience Center (iNANO) Aarhus University DK‐8000 Aarhus C Denmark; ^3^ Department of Chemistry University of Nebraska‐Lincoln NE 68588 Lincoln USA; ^4^ Beijing Advanced Innovation Center for Materials Genome Engineering State Key Laboratory for Advanced Metals and Materials University of Science and Technology Beijing Beijing 100083 China; ^5^ Key Laboratory of Colloid and Interface Chemistry Ministry of Education Shandong University Jinan 250100 China; ^6^ State Key Laboratory of Electronic Thin Films and Integrated Devices University of Electronic Science and Technology of China Chengdu 610054 China; ^7^ Department of Chemical and Biomolecular Engineering and Department of Mechanical and Materials Engineering University of Nebraska‐Lincoln NE 68588 Lincoln USA

**Keywords:** ambipolar carrier, density function theory, electrochemical microcells, hydrogen evolution, model catalysis

## Abstract

An improved understanding of the origin of the electrocatalytic activity is of importance to the rational design of highly efficient electrocatalysts for the hydrogen evolution reaction. Here, an ambipolar single‐crystal tungsten diselenide (WSe_2_) semiconductor is employed as a model system where the conductance and carrier of WSe_2_ can be individually tuned by external electric fields. The field‐tuned electrochemical microcell is fabricated based on the single‐crystal WSe_2_ and the catalytic activity of the WSe_2_ microcell is measured versus the external electric field. Results show that WSe_2_ with electrons serving as the dominant carrier yields much higher activity than WSe_2_ with holes serving as the dominant carrier even both systems exhibit similar conductance. The catalytic activity enhancement can be characterized by the Tafel slope decrease from 138 to 104 mV per decade, while the electron area concentration increases from 0.64 × 10^12^ to 1.72 × 10^12^ cm^−2^. To further understand the underlying mechanism, the Gibbs free energy and charge distribution for adsorbed hydrogen on WSe_2_ versus the area charge concentration is systematically computed, which is in line with experiments. This comprehensive study not only sheds light on the mechanism underlying the electrocatalysis processes, but also offers a strategy to achieve higher electrocatalytic activity.

## Graphical abstract

An ambipolar single‐crystal tungsten diselenide (WSe_2_) semiconductor electrocatalyst is employed as a benchmark system for interfacial model catalysis. By tuning the external electric field, the conductance and carrier of ambipolar WSe_2_ can be individually modulated. Combining the experimental and theoretical study, it is demonstrated that the electron carrier plays a key role in the hydrogen evolution reaction.

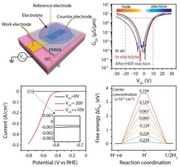

## 1 Introduction

Fossil fuel, as a dominant energy supply, gives rise to environmental pollution and leads to climate change. Therefore, the development of clean and renewable energy is the key way to meet the increasing global energy requirement and to resolve the environmental pollution caused by the overuse of fossil fuels.^1^, ^2^, ^3^ Hydrogen has been considered as a promising green energy carrier due to its highest energy density and pollution‐free and carbon‐free production.^4^, ^5^, ^6^ Generally, the splitting of water by electrocatalysts is regarded as a promising way for hydrogen generation.^7^ The key to realizing this reaction is to find an efficient and robust electrocatalyst which can effectively lower the reaction barrier and result in highly efficient utilization of electric.^8^

One key reaction in water splitting is the hydrogen evolution reaction (HER),^5^, ^9^, ^10^ which includes three reactions:

(1) $$\mathrm{Volmer}\;\mathrm{reaction}\;:\;H_{3}O^{+}+\mathrm{site}+e^{-}\to \mathrm{site}-H_{\mathrm{ads}}+H_{2}O$$

(2) $$\mathrm{Heyrovsky}\;\mathrm{reaction}\;:\;\mathrm{site}-H_{\mathrm{ads}}+H_{3}O^{+}+e^{-}\to H_{2}+H_{2}O+\mathrm{site}$$

(3) $$\mathrm{Tafel}\;\mathrm{reaction}\;:\;\mathrm{site}-H_{\mathrm{ads}}+\mathrm{site}-H_{\mathrm{ads}}\to H_{2}+2\mathrm{site}$$

There are two pathways for generating molecular hydrogen, following either Volmer–Heyrovsky reaction or Volmer–Tafel reaction.^11^ Therefore, the Volmer reaction is the key step in HER and it is strongly dependent on the electron transfer, the density of active sites, and the Gibbs free energy of adsorbed atomic hydrogen.^12^, ^13^, ^14^, ^15^ To improve these factors, many strategies have been developed, such as improving the catalyst's conductance by introducing a conductive network, increasing the number of active sites by making nanostructure, and modifying materials through element doping or compositing.^3^, ^4^, ^7^, ^16^, ^17^, ^18^ Among them, the enhancement mechanism through improving conductivity is still unclear, possibly due to the complicated model system with varying factors like active site or nanostructures. Recently, it has been reported that reducing the resistance of catalyst by phase changing or introduction of external field can enhance the catalytic activity as both strategies can facilitate electron injection onto the active sites.^19^, ^20^, ^21^ Because the external electric field can modify the resistance of semiconductor and the carrier type and concentration, it is hard to distinguish whether the enhanced HER performance is due to enhanced conductance or due to increased electron concentration. Revealing this mechanism not only can further help to understand the enhancement mechanism, but also can help to rationally design of new electrocatalyst with high activity.

However, distinguishing the role of conductance and carrier on the powder‐like catalyst is very complicated due to factors such as the uncontrolled defects, nanostructures, and conductance. As an alternative, the high‐quality single crystal flake is an ideal model electrocatalyst for fundamental study.^22^ Although the conductance of unipolar semiconductor (n‐type or p‐type semiconductor) can be tuned by external electric field,^23^ the dominant carrier type could hardly switch between electron and hole. In this study, the ambipolar single‐crystal WSe_2_ semiconductor is employed as a model electrocatalyst because the conductance of WSe_2_ could be effectively tuned, and more importantly, its dominant carrier can be easily switched between hole and electron by external electric field.^24^, ^25^ Therefore, the influence on catalytic activity by conductance or by carrier can be effectively isolated. The ambipolar WSe_2_‐based electrochemical microcell is constructed. By tuning the external electric field, the catalytic activity can be in situ estimated under the same conductance with the opposite carrier type. This is a good platform to investigate the role of conductance and carrier in the catalytic reaction. The results show that with the similar conductance, WSe_2_ with electrons as the dominant carrier exhibits much higher catalytic activity than that of WSe_2_ with holes as the dominant carrier. With increasing electron concentration, the catalytic activity is enhanced, as demonstrated by the decrease of Tafel slope from 138 to 104 mV per decade and the decrease of overpotential at 10 mA cm^−2^ from 0.37 to 0.28 V. These results directly demonstrates that the electron carrier concentration plays a more important role in HER. Further, the WSe_2_ with different area charge concentration has been studied by using density‐functional‐theory (DFT) computation. The Gibbs free energy and the charge distribution have been computed, both being in line with our experiment.

## 2 Results and Discussion

**Figure 1:**
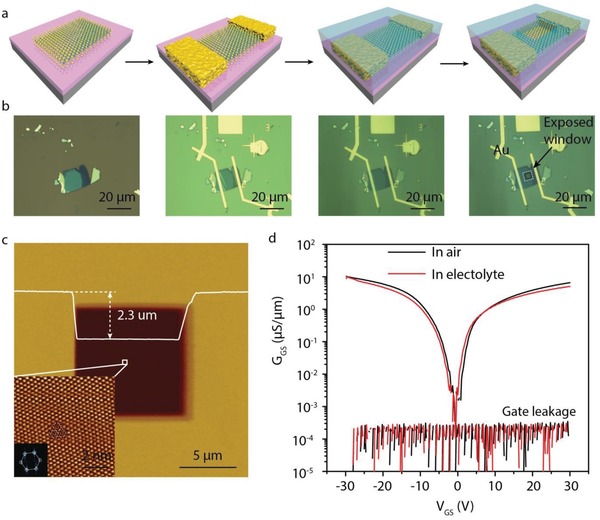
The device fabrication on WSe_2_ basal plane. a) A schematic diagram of the fabrication process and b) a realistic optical image of WSe_2_ device. c) The AFM height image of the protected PMMA layer, where the window is exposed by EBL only on the WSe_2_ basal plane. The inset shows the low pass filtered atomic pattern acquired from the center region by lateral force microscopy. d) The electrical performance of WSe_2_ device in the air and an electrolyte. The electrolyte is 0.5 mol L^−1^ H_2_SO_4_ solution.

The WSe_2_ flake was transferred on SiO_2_/Si substrate by mechanical exfoliation. The thickness of WSe_2_ was identified according to the optical contrast and AFM morphology measurement. Our previous study demonstrates that the electrical property of WSe_2_ is strongly dependent on its thickness, and the WSe_2_ flake with 12 layers has the best ambipolar electrical property with the highest carrier mobility.^24^ Therefore, in this study, the WSe_2_ flakes with a thickness of 12 layers were selected. The standard electron beam lithography process and followed electron beam evaporation were carried to form the electrical connections. To archive enough space to form an electrolyte droplet, the electrodes are extended to more than 1 cm, locating near the edge of the substrate. Then, PMMA (A11 concentration) was coated and expose the reaction window on the WSe_2_ flake by electron beam lithography. It should be noted that the surface of the sample has been fully covered by PMMA except the exposed window. The fabrication schema and corresponding optical images are shown in **Figure** **1**a,b. Figure 1c displays the AFM morphology of the exposed window, where the thickness of the covered PMMA is about 2.3 µm. The high quality of the WSe_2_ can be demonstrated by the atomic pattern inset in Figure 1c which is required by lateral force microscopy. The micro‐size topographic AFM image is shown in Figure S1a (Supporting Information). There are no PMMA residues and no discontinuities (edges or steps) on the WSe_2_ surface, indicating that the catalytic activity should stem from WSe_2_ basal plane, rather than from the discontinuities (edges or steps) or the defects. Figure 1d shows the transfer characterization of the WSe_2_ transistor where the gate voltage is giving through the back conductive silicon. It is clearly seen that the WSe_2_ transistor exhibits the ambipolar transport behavior, where the hole is the dominant carrier for *V*_GS_ < 0 V and the electron is the dominant carrier for *V*_GS_ > 0 V.^24^ Further, the transport curve shows a symmetric characterization in both hole and electron branches, indicating the balanced hole and electron conductance. After introducing the 0.5 m H_2_SO_4_ electrolyte, the source‐drain conductance and the gate leakage as a function of gate voltage have no obvious change, suggesting WSe_2_ is stable in the electrolyte showing symmetric conductance. The ambipolar transport behavior of WSe_2_ is a platform to study the role of the carrier in surface catalysis since WSe_2_ could have a similar conductance but with opposite carrier type.

**Figure 2:**
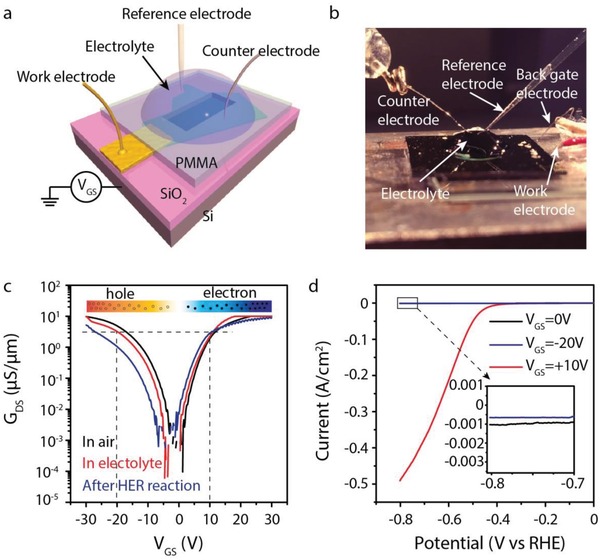
The electrocatalytic performance in electron and hole branches on the ambipolar WSe_2_ device. a) A schematic of the setup showing a single gold pad connected with a WSe_2_ flake used as the working electrode. b) Photography of the electrochemical microcell. c) The electrical transfer properties of the WSe_2_ device in the air, in the electrolyte and after HER reaction, respectively. d) Typical polarization curves measured for the WSe_2_ device when the gate voltage is 0, −20, and +10 V, respectively. The electrocatalytic measurement is in 0.5 m H_2_SO_4_ with a scan ratio of 5 mV s^−1^. The inset is the corresponding Tafel plot.

To explore the role of the carrier in surface catalysis, the ambipolar WSe_2_ transistor with symmetric conductance was employed to construct an electric field tuned electrochemical microcell, as shown in **Figure** **2**a. In this setup, the surface catalysis was assessed by measuring the water splitting property through HER with employing the WSe_2_ as the electrocatalyst. HER is carried out using a three‐electrode configuration with Pt wire as the counterelectrode, micro‐Ag/AgCl electrode as the reference electrode, and the electrode connected with the WSe_2_ flake as the working electrode.^26^ Figure 2b shows the real image of the setup. The transfer curves of the WSe_2_ transistor at different conditions (in the air, in the electrolyte and after HER) are shown in Figure 2c. The HER polarization curves (Figure 2d) were measured at different gate voltage corresponding to different carrier envelope. As seen, the WSe_2_ flake exhibits a similar conductance (≈3 µS µm^−1^) at *V*_GS_ = +10 and −20 V but with electron and hole as a dominant carrier, respectively. It is surprising that the polarization current at *V*_GS_ = +10 V can reach about 0.5 A cm^−2^ (when the potential is −0.8 V vs RHE), which is three orders higher than the polarization current at *V*_GS_ = −20 V. This implies that the conductance of catalysts may not be the key factor in catalytic reaction. The lower polarization current at *V*_GS_ = 0 V is much lower since WSe_2_ is an insulator. The blue line in Figure 2c shows the transport curve after these HER measurements which directly confirm the stability of WSe_2_, further confirming that the WSe_2_ flake at *V*_GS_ = +10 and −20 V exhibits the similar conductance but different carrier type. Considering that the solution gate from the counterelectrode has little influence on the electrochemical catalytic reaction,^21^ the enhanced catalytic activity should be originated from the material's property. The topographic AFM image and Raman spectra of WSe_2_ prior to and after the reaction is shown in Figure S1 (Supporting Information). As seen, there are no obvious changes in morphology and Raman spectra, indicating that the WSe_2_ basal plane is stable.

**Figure 3:**
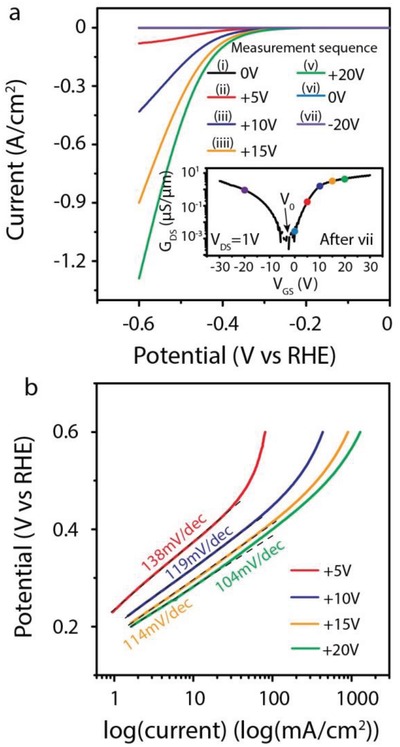
Gate voltage‐dependent electrocatalytic activity on the ambipolar WSe_2_ device. a) The polarization curves measured under different gate voltages, and the inset shows the electrical transport property of WSe_2_ device after all measurements. b) The Tafel plots from (a).

The polarization curves of the WSe_2_ flake at different gate voltages are shown in **Figure** **3**. The gate voltages are tuned as the sequence of 0, +5, +10, +15, +20, 0, and −20 V. The polarization curves of *V*_GS_ = 0 V are overlapped excluding the variation of WSe_2_ material during the measurement. After all HER measurements, the transfer curve of the WSe_2_ transistor in electrolyte still exhibits the symmetric conductance character, meaning the above HER measurements are performed under symmetric transport condition. The carrier concentration could be calculated by *n* = *C*_0_(*V*_GS_ − *V*_0_)/*e*, where *n* is the carrier concentration, *C*_0_ is the capacity, *V*_0_ is the neutral point, and *e* is the electron charge.^27^ In this device, the *V*_0_ is about −4 V, possible due to the intrinsic dopant in the WSe_2_ crystal. Figure 3b shows the Tafel curves which are fitted according to Tafel equation: η = *b* log(*j*) + *a*, where η is the potential, *b* is the Tafel slope, and *j* is current density, respectively. The carrier area concentration, conductance, Tafel slope, and overpotential are summarized in **Table** **1**. As seen, the polarization current is largely boosted with the increasing of gate voltage from 0 to +20 V. The overpotentials calculated at the polarization current of 10 mA cm^−2^ are 0.37, 0.32, 0.30, and 0.28 V when gate voltages are +5, +10, +15, and +20 V, respectively. Even WSe_2_ at *V*_GS_ = −20 V exhibits a higher conductance (0.932 µS µm^−1^) compared with WSe_2_ at *V*_GS_ = +5 V (conductance is 0.174 µS µm^−1^), the polarization current at *V*_GS_ = −20 V is still much lower than that at *V*_GS_ = +5 V, which shows a similar phenomenon with the previous device in Figure 2. During tuning the gate voltage, the carrier concentration and also the conductance are both tuned. However, the conductance of WSe_2_ increases from 0.174 to 4.695 µS µm^−1^ when the gate voltage increases from +5 to +20 V. Considering that when the resistance is smaller than 10–100 kΩ mm (corresponding to that conductance is higher than 0.01–0.1 µS µm^−1^), the variation of resistance or conductance gives no obvious influence on catalytic activity.^20^, ^26^ Thus, we can propose that the varied catalytic activity (Tafel slope or overpotential) is due to the increased area electron concentration rather than the improved conductance. The results also suggest that the electron carrier is the key factor during electrocatalytic reaction rather than the conductivity, and that synthesis of electron‐rich electrocatalyst is preferred. Although the external field modulation may not lead to the highest catalytic performance, combination with other strategies, e.g., introducing point defects via doping heteroatoms^28^, ^29^ may further enhance the catalytic performance.

**Table 1:** The carrier concentration (per unit area), conductance, Tafel slope, and overpotential of WSe_2_ electrocatalysts under different gate voltage

*V*_GS_ [V]	*N* [×10^12^ cm^−2^]	*G* [µS µm^−1^]	Tafel slope [mV per decade]	Overpotential (at 10 mA cm^−2^, V vs RHE)
0	−0.29	2.393e−3	–	–
5	−0.64	0.174	138	0.37
10	−1.00	1.595	119	0.32
15	−1.36	3.271	114	0.30
20	−1.72	4.695	104	0.28
−20	+1.15	0.932	–	–

“−”: electron carrier; “+”: hole carrier.

**Figure 4:**
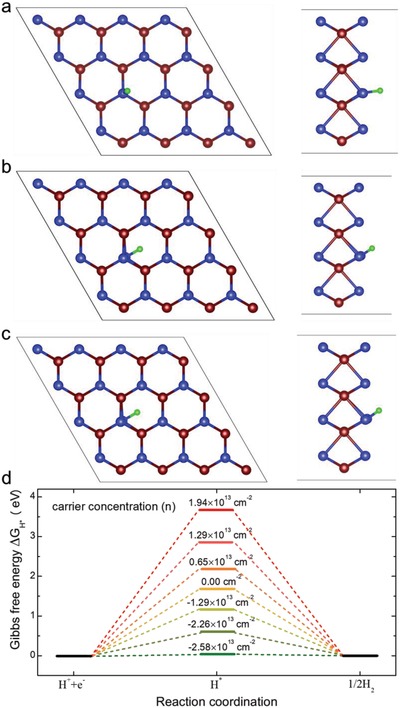
DFT calculation results of hydrogen atom adsorbed on charged WSe_2_ sheet with respect to the different carrier concentration (per unit area *n*). The associated adsorption sites at *n* = a) 0.65 × 10^13^ cm^−2^, b) neutral, and c) −2.58 × 10^13^ cm^−2^. d) The Gibbs free‐energy diagram of hydrogen evolution reaction. The blue, purple and green balls represent Se, W, and H atoms, respectively. The *n* > 0 corresponds to the added hole in the system, while *n* < 0 corresponds to the added electron in the system.

The in situ HER measurements are all made under the same condition, for example, the same contact property and the same surface structure. The experimental results indicate that WSe_2_ with electrons as the dominant carrier exhibits much higher catalytic activity than that of WSe_2_ with holes as the dominant carrier when they have similar conductance. To further understand the underlying mechanism of enhanced HER activity by extra electron or hole, DFT calculation is carried out to evaluate the Gibbs free energy of a hydrogen atom on a charged WSe_2_ sheet, a known factor for determining the HER activity. For seeking a starting configuration of the most stable adsorption site, we examine all possible initial positions for H adsorption with symmetry considerations. Figure S2 (Supporting Information) depicts the top and side views of three optimized configurations. The corresponding formation energy is calculated by $E_{\mathrm{formation}}=E_{{\mathrm{H+WSe}}_{2}}-E_{H}-E_{{\mathrm{WSe}}_{2}}$, where $E_{{\mathrm{H+WSe}}_{2}}$ is the total energy of the H atom adsorbed on the WSe_2_ sheet, *E*_H_ is the energy of the H atom, and $E_{{\mathrm{WSe}}_{2}}$ is the energy of the WSe_2_ sheet. As seen, the hydrogen atom adsorbed on the top of six‐ring and near the Se atom (Figure S2c, Supporting Information) is the favorable energy state with the lowest formation energy. Based on the most stable adsorption structure, **Figure** **4**a–c shows the optimized WSe_2_ sheet with an adsorbed H at different carrier area concentration (*n*) of 6.45 × 10^13^, 0.00, and −2.58 × 10^13^ cm^−2^, respectively. The negative carrier means the carrier is an electron, while the positive carrier means the carrier is a hole. The corresponding carrier concentration varies from 1.94 × 10^13^ to −2.58 × 10^13^ cm^−2^, calculated based on the area of the model system (1.55 × 10^−14^ cm^2^). When the extra carrier of the system is zero, the preferential H position is near a Se atom, where a Se—H bond is formed. Interestingly, the position of the adsorbed H with respect to the Se atom depends on its charge state. In the neutral and negative charge states, the H is located at the antibonding site, as shown in Figure 4b,c. The Se—H bond is aligned with a W—Se bond, pointing toward the surface spacing.

The preferential position of the adsorbed H in the positive charge state is different, where the adsorbed H moves toward the top of a Se atom (Figure 4a). A similar adsorption site of H was reported in MoS_2_.^28^, ^29^ Generally, a good HER catalyst should feature $\left|\Delta G_{H^{*}}\right|$ value near zero, which allows optimal adsorption/desorption kinetics. Further atomic insight into the carrier‐induced HER activity differences is obtained from the computed adsorption Gibbs free energy of the bonded hydrogen atom. The calculated free energy is illustrated in Figure 4d. In the cases of the extra holes, the $\Delta G_{H^{*}}$ values are too positive, and the H* species cannot be adsorbed onto the catalyst surface, and thus they are also unsuitable for HER. Compared with the pristine WSe_2_ structure without an extra carrier, the extra electrons lead to more negative $\Delta G_{H^{*}}$ values, indicating a more favorable adsorption process for the H* species. When the extra electron area concentration is −2.58 × 10^13^ cm^−2^, the catalyst exhibits a desired $\Delta G_{H^{*}}$ value of 0.033 eV, which is closer to zero than that of the well‐known Pt‐based catalyst ($\left|\Delta G_{H^{*}}\right|$ = 0.09 eV).^30^, ^31^, ^32^ The basic trend of our theoretical results is qualitatively consistent with that of experimental results, even though the specific values are not exactly the same. We examine the effects of spin polarization and the use of different pseudopotential in our DFT calculations. As shown in Figures S4 and S5 (Supporting Information), neither the spin nor the GBRV pseudopotential can have much influence on the variation trends of the free energy, indicating the reliability of the current DFT calculation results. To gain a deeper understanding of the experimental results, we also examine the role of defect sites in the catalytic reaction (see Figures S6 and S7 in the Supporting Information). Both W point defects and edge defects can decrease the Gibbs free energy to a certain level, depending on the position relative to the defect site, whereas the Se vacancy can increase the Gibbs free energy. The opposing effects of the defects on the Gibbs free energy suggest that the defects alone cannot explain the experimental results of increased HER activity. Alternatively, we find that the HER activity is very localized to the adsorption site, suggesting that the system area in the DFT calculation plays a decisive role in the electron area concentration. Besides the system size of 4 × 4 × 1 supercell, we also consider a larger supercell size with 8 × 8 × 1 unit cells (193 atoms) to illustrate this point. As shown in Figure S8 (Supporting Information), the larger supercell results in reduced electron area concentration to nearly one eighth. It is known that Gibbs free energy Δ*G*_H*_ is a commonly used descriptor for characterizing the catalytic activity of H atom adsorption. Some other important descriptors for understanding the structure‐activity‐selectivity relationships include the electronic level, i.e., electron charge, valance band position, etc.^28^

**Figure 5:**
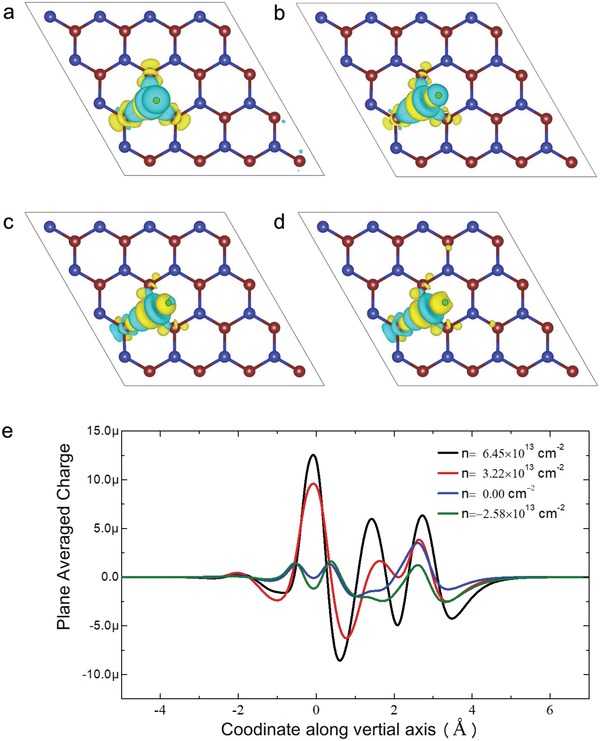
The charge difference plot of the hydrogen atom adsorbed on the charged WSe_2_ sheet with different carrier concentration (per unit area *n*): a) *n* = 6.45 × 10^13^ cm^−2^; b) *n* = 3.22 × 10^13^ cm^−2^; c) *n* = 0.00 cm^−2^; and d) *n* = −2.58 × 10^13^ cm^−2^. The isovalue is 0.0025 e Bohr^−3^. The yellow region indicates electron accumulation, and the cyan region indicates electron depletion, respectively. The H atom is highlighted by a black circle. e) The plane averaged carrier concentration along the out‐of‐plane direction when the WSe_2_ has a different charge density.

The difference in the H atomic position and the interaction between H atom and the WSe_2_ sheet cause distinct charge transfer behaviors. To correlate this charge transfer behavior with the HER activity, we construct the charge‐difference plot, where the charge difference is calculated by $\Delta \rho =\rho_{{\mathrm{H+WSe}}_{2}}-\rho_{H}-\rho_{{\mathrm{WSe}}_{2}}$. Here, $\rho_{{\mathrm{H+WSe}}_{2}}$ is the total charge density of the H atom adsorbed on the WSe_2_ sheet, ρ_H_ is the charge density of the H atom, and $\rho_{{\mathrm{WSe}}_{2}}$ is the charge density of the WSe_2_ sheet.^33^ **Figure** **5**a–d present the WSe_2_ sheet adsorbed with one H atom with respect to different carrier area concentration 6.45 × 10^13^, 3.22 × 10^13^, 0.00, and −2.58 × 10^13^ cm^−2^, respectively. Similar to the reported MoS_2_ for HER process,^16^, ^26^, ^34^ because of the very high level of 3p orbital with respect to the H 1s orbital, the H adsorption on the basal plane of pristine WSe_2_ is too weak ( $\Delta G_{H^{*}}$= 1.68 eV), leading to a poor HER performance. As shown in Figure 5a–d, when the extra carrier is a hole, the hydrogen atom is surrounded by the electron depletion region, as denoted by Figure 4e, which is unfavorable for HER activity. With the extra electron increasing from 0.00 to −2.58 × 10^13^ cm^−2^, it will strengthen the adsorption and change the charge state of H atom from electron depletion to electron accumulation, which offsets the energy level for enhancing the H adsorption and HER activity. However, with the area concentration of extra electron further increasing, the further enhanced adsorption energy would render the desorption process difficult, and thus deteriorate the HER performance. In other words, the extra electron carrier would contribute to the charge redistribution with the decrease of adsorption energy. A suitable amount of electrons would lead to favorable adsorption/desorption of H atoms for high‐performance HER. To illustrate in detail the nature of charge transfer behavior, the plane averaged carrier concentration along the out‐of‐plane direction is shown in Figure 5e. The plane averaged carrier concentration is calculated by $\Delta \sigma (z)=\sigma_{{\mathrm{H+WSe}}_{2}}(z)-\sigma_{H}(z)-\sigma_{{\mathrm{WSe}}_{2}}(z)$, where $\sigma_{{\mathrm{H+WSe}}_{2}}(z)$ is plane averaged charge density of the combined H–WSe_2_ system, σ_H_(*z*) and $\sigma_{{\mathrm{WSe}}_{2}}(z)$ are, respectively, the plane averaged charge density of the isolated H atom and WSe_2_ layer, which are calculated by fixing the atomic positions of the corresponding components in the combined system. The positive and negative values indicate charge loss and gain, respectively. It is clear that the extra electron contributes to a smoother distribution of charge, which attributes to the higher mobility of extra electron. However, the hole carrier causes a more localized charge transfer. Both the charge depletion and charge accumulation constitute the charge redistribution behavior, and ultimately, affect the hydrogen adsorption. The DFT calculation demonstrates that the hole carrier or overly introduced electron carrier can induce too positive or too negative Gibbs free energy, neither favorable to the adsorption and desorption of hydrogen atom. An appropriate area concentration of electron carrier would give rise to optimal Gibbs free energy, closer to the value of Pt.

## 3 Conclusion

In summary, the role of the carrier on catalytic activity has been systemically studied from experiment and DFT calculation. The ambipolar WSe_2_‐based electrochemical microcell provides the platform to estimate the in situ catalytic activity under the same condition (defect, active site, nanostructure, etc.). With varying the external field, the dominant carrier in WSe_2_ can switch between electron and hole, while the conductance of WSe_2_ can be tuned as well. The effect of the electron carrier, hole carrier, and conductance in catalysis reaction has been carefully discussed. The results show that with the similar conductance, WSe_2_ with the electrons as the dominant carrier shows much higher catalytic activity than that with the holes as the dominant carrier. With increasing the gate voltage in electron dominated WSe_2_, the catalytic activity can be further enhanced with the Tafel slope decreasing from 138 to 104 mV per decade, attributing to the increased electron concentration rather than the conductance. Furthermore, the Gibbs free energies of adsorbed atomic hydrogen on WSe_2_ with different electron and hole area concentrations are calculated. The results show that $\Delta G_{H^{*}}$ of electron dominated WSe_2_ is much lower than that of hole dominated WSe_2_. Moreover, the $\Delta G_{H^{*}}$ can be lowered to 0.033 from 1.68 eV by injecting electrons into pristine WSe_2_ with an electron area concentration of 2.58 × 10^13^ cm^−2^. The results show that the electron carrier plays a more important role in HER, rather than the conductance, which provides a new strategy to rationally design highly efficient electrocatalysts.

## 4 Experimental Section

*Device Fabrication and Measurement*: The WSe_2_ flakes were mechanically exfoliated from a bulk crystal onto a SiO_2_/Si substrate with a size of 2 cm × 2 cm. The WSe_2_ flake with a thickness of 12 layers, which exhibits the highest carrier mobility and ambipolar behavior,^24^ is selected. The source and drain electrodes were patterned with electron beam lithography. Then 10 nm Ti/40 nm Au layers were evaporated by electron beam deposition with a depositing speed of 0.2 Å s^−1^. Before evaporation, the sample was kept overnight under the high vacuum in the electron beam deposition system. To have enough space to form an electrolyte droplet, the source and drain electrodes were extended to near the edge of the substrate with a length of about 1 cm. A second electron beam lithography process was employed to expose the basal plane of WSe_2_. To fully cover the WSe_2_ edges and the electrodes, PMMA with a concentration of 11% in anisole was spin‐coated on the sample and then a conductive polymer was coated on its surface to eliminate the charge effect during electron beam lithography. More attention was devoted to ensuring that no gold electrode and WSe_2_ edge were exposed to the electrolyte. After opening the window on the WSe_2_ flake, the sample was baked at 180 °C for 10 min. The electrical transport property of WSe_2_ transistor was carried by Keithley 4200 SCS, and the field tuned electrochemical reaction was performed on electrochemical workstation combined with Keithley 4200 SCS. Linear sweep voltammetry with a scan rate of 5 mV s^−1^ was conducted in 0.5 m H_2_SO_4_ solution, using a Pt wire as the counterelectrode. The micro‐reference electrode was calibrated for the reversible hydrogen potential, where *E*(vs RHE) = *E*(vs Ag/AgCl) + 0.197 V. Before measurement, the H_2_SO_4_ solution was degassed using pure Ar gas.

*Computation*: DFT calculations were carried out using the Hartwigsen–Goedeker–Hutter norm‐conserving pseudopotentials^35^ and the Perdew–Burke–Ernzerhof (PBE) exchange‐correlation functional^36^ as implemented in the Quantum ESPRESSO package.^37^, ^38^ The spin‐polarized calculation was employed,^39^ and the vdW + DF2 functional was selected to account for van der Waals (vdW) interactions.^40^ The model system is a 4 × 4 supercell of WSe_2_ (with 48 atoms) and a single hydrogen atom adsorbed on the surface. In the direction vertical to the surface, the supercell extends with a vacuum space for 30 Å. The Brillouin zone is sampled using a 3 × 3 × 1 Monkhorst–Pack *k*‐point grid. Atomic positions are optimized until the maximum force on all atoms is less than 0.001 a.u. Cutoffs of 40 and 160 Ry were chosen for the wave function and the electronic density, respectively. For the computational scheme based on pseudopotential and periodic slab geometry, it is challenging to deal with the charged system when imposing the periodic boundary condition in the surface normal direction.^41^, ^42^ In the case of the system with the extra carrier, an effective screening medium method (ESM)^42^, ^43^ was utilized to treat effectively charged slabs. ESM screens the electronic charge of a polarized/charged medium along one perpendicular direction by introducing a classical charge model and a local relative permittivity into the DFT calculation framework. In this condition, excess or deficit charge is accommodated on one side of a slab, and the image charge is automatically induced in the medium. Thus, this permits calculation by using open boundary conditions. Lastly, for DFT calculation with a larger supercell with 8 × 8 × 1 unit cells (193 atoms), the Gamma point was adopted.

## Conflict of Interest

The authors declare no conflict of interest.

## Supporting information

Supporting Information
