# Supplementary material for: Reversing Interfacial Catalysis of Ambipolar WSe2 Single Crystal
Source: Adv Sci (Weinh). 2019 Dec 5;7(3):1901382. doi: 10.1002/advs.201901382 (PMC7001631; doi:10.1002/advs.201901382)
Supplement: Supplementary file 1 — Supporting Information [file ADVS-7-1901382-s001.pdf]

Copyright WILEY-VCH Verlag GmbH & Co. KGaA, 69469 Weinheim, Germany, 2019.

ADVANCED SCIENCE
Open Access

Supporting Information

for *Adv. Sci.*, DOI: 10.1002/advs.201901382

Reversing Interfacial Catalysis of Ambipolar WSe_2 Single Crystal

Zegao Wang, Hong-Hui Wu, Qiang Li, Flemming Besenbacher, Yanrong Li, Xiao Cheng Zeng, and Mingdong Dong**

WILEY-VCH

Copyright WILEY-VCH Verlag GmbH & Co. KGaA, 69469 Weinheim, Germany, 2019.

Supporting Information

Reversing Interfacial Catalysis of Ambipolar WSe_2 Single Crystal

Zegao Wang, Hong-Hui Wu, Qiang Li, Flemming Besenbacher, Yanrong Li,

Xiao Cheng Zeng, Mingdong Dong**

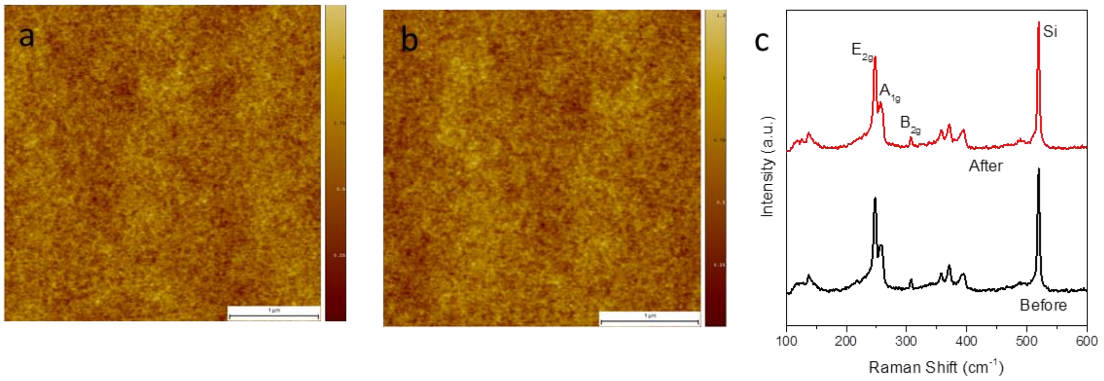

Figure S1. (a) and (b) The topographic AFM image of WSe_2 surface before and after the electrocatalytic reaction. (c) The Raman spectra of WSe_2 before and after the electrocatalytic reaction. As seen, there is no PMMA residues and no discontinuities (edges or steps) on WSe_2 surface in the micro-scale. Combined with the atomic pattern (Figure 1c), we can propose that the electrocatalytic property originates from the WSe_2 basal plane, rather than the discontinuities (edges or steps) or the defects. Considering the no obvious change after electrocatalytic reaction from topographic AFM image and Raman spectra, we believe that the WSe_2 basal plane is stable during the reaction.

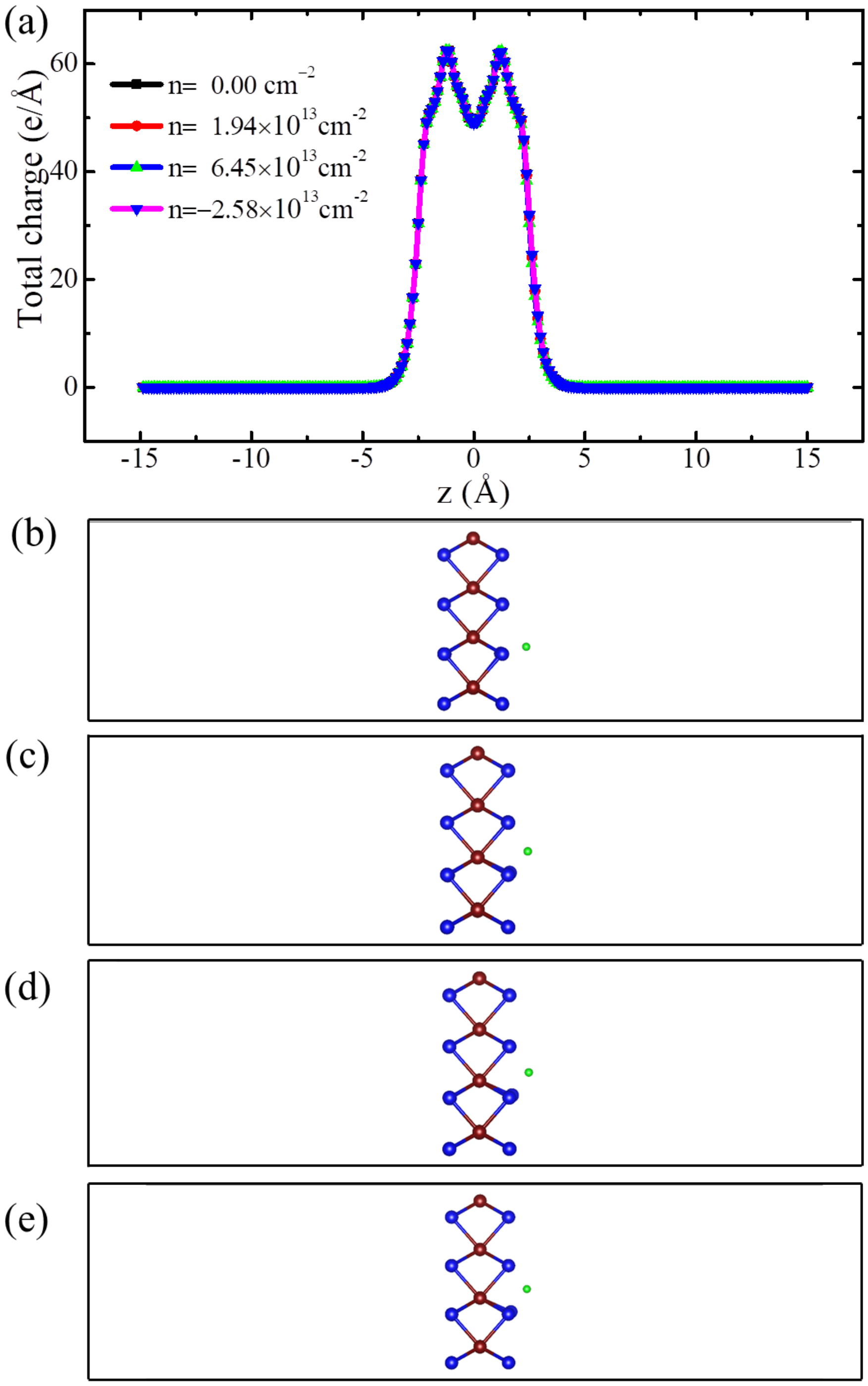

Figure S2. Calculation results by ESM model. (a) The total charge along z direction, the side profile of the net charge concentration (per unit area) (b) $n=6.45\times10^{13}$ cm^{-2}, (c) $n= 1.94\times10^{13}$ cm^{-2}, (d) $n=0.00$ cm^{-2} and (e) $n= -2.58\times10^{13}$ cm^{-2}.

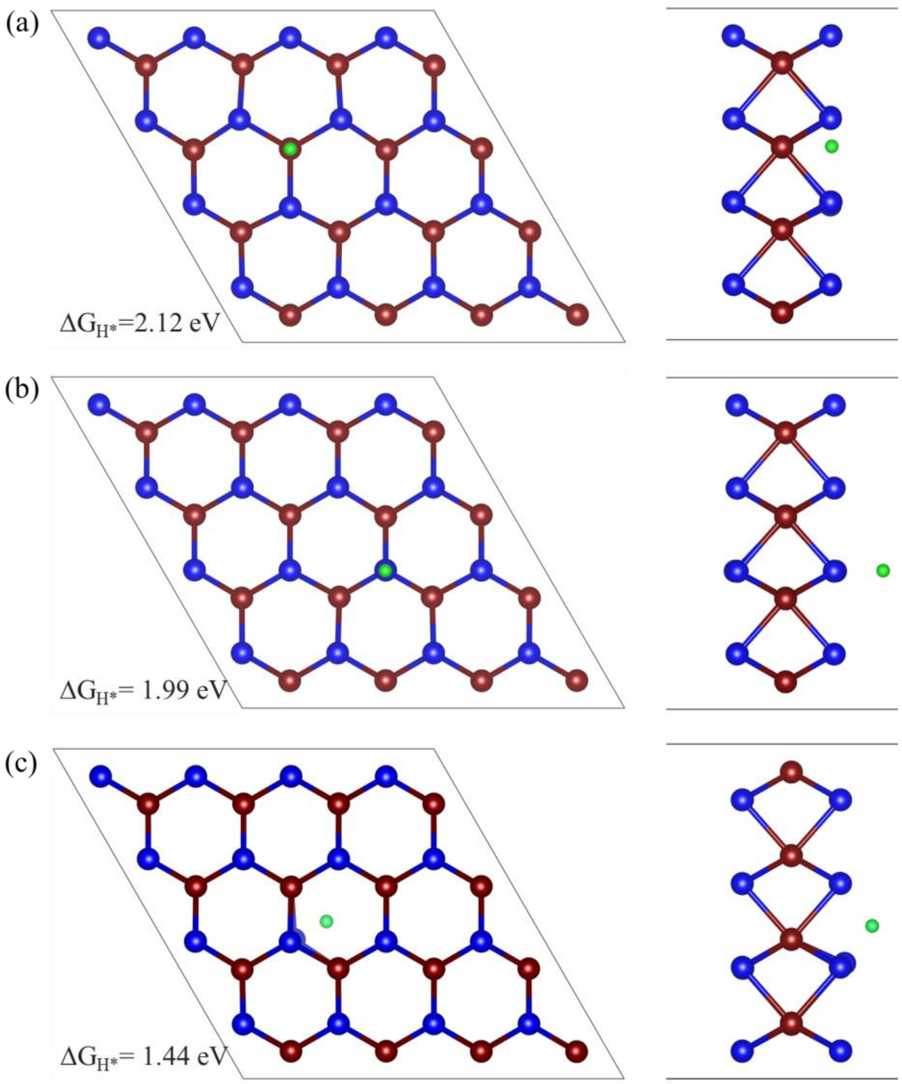

Figure S3. Three configurations and the associated adsorption energy of the H atom on the WSe_2 sheet. The blue, dark red, cyan balls represent Se, W, and H atoms, respectively.

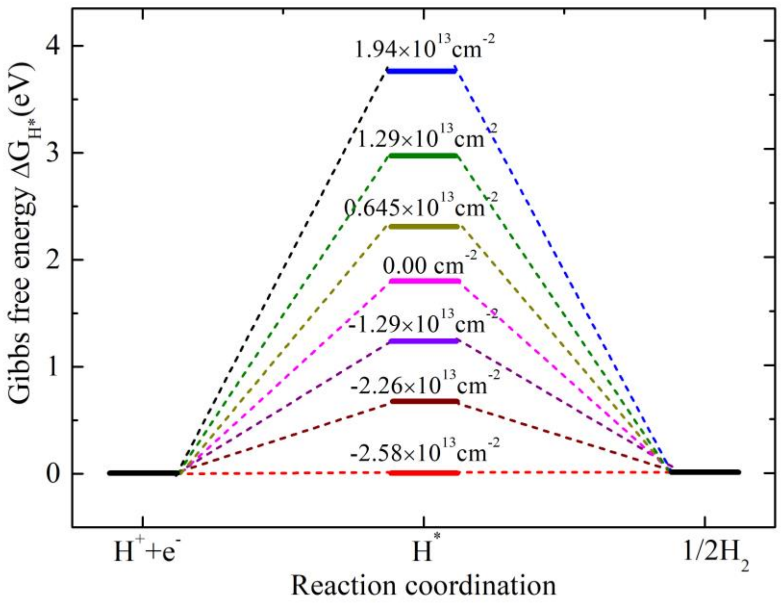

Figure S4. The calculated Gibbs free energy without spin at different carrier concentration (per unit area).

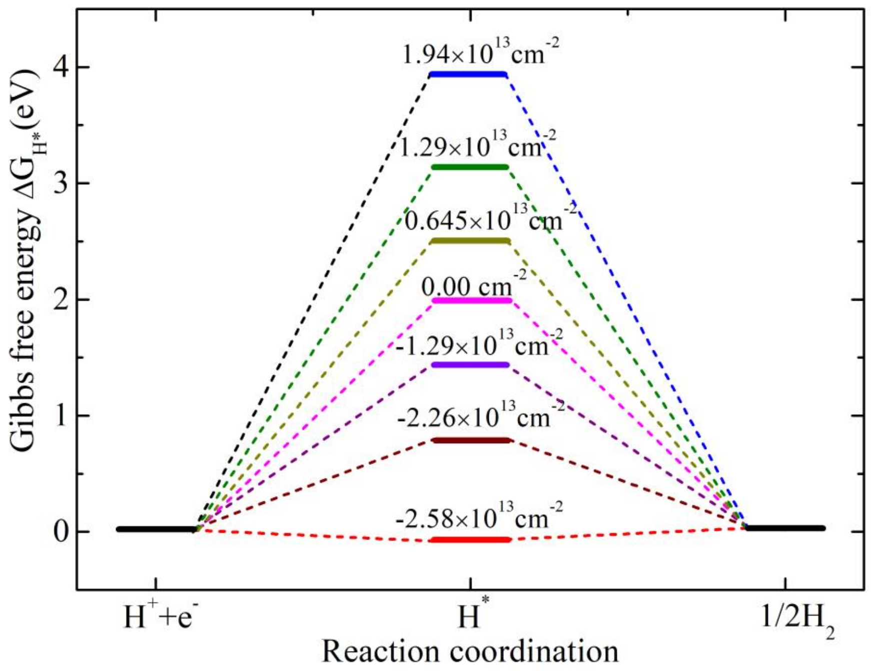

Figure S5. The Gibbs free energy calculated with GBRV pseudopotential at different carrier concentration (per unit area).

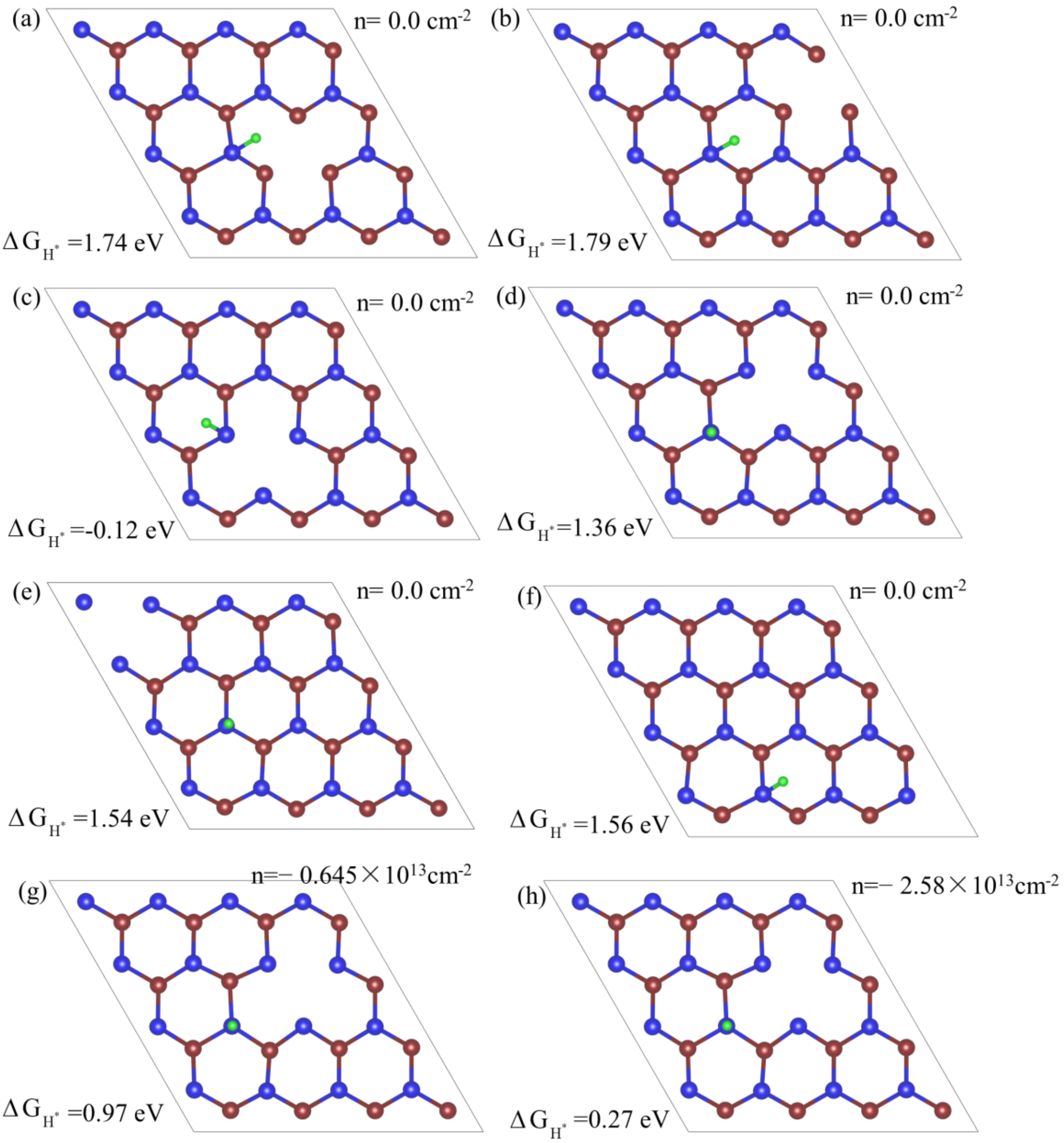

Figure S6. The effect of a point defect on the Gibbs free energy: Different adsorption sites for (a)-(b) the optimized model with Se vacancy, (c)-(f) the optimized model with W vacancy; the effect of charge carrier concentration (per unit area) n on the Gibbs free energy of the model (d), (g) n= -0.645×10^{13} cm^{-2}, (h) n= -2.58×10^{13} cm^{-2}.

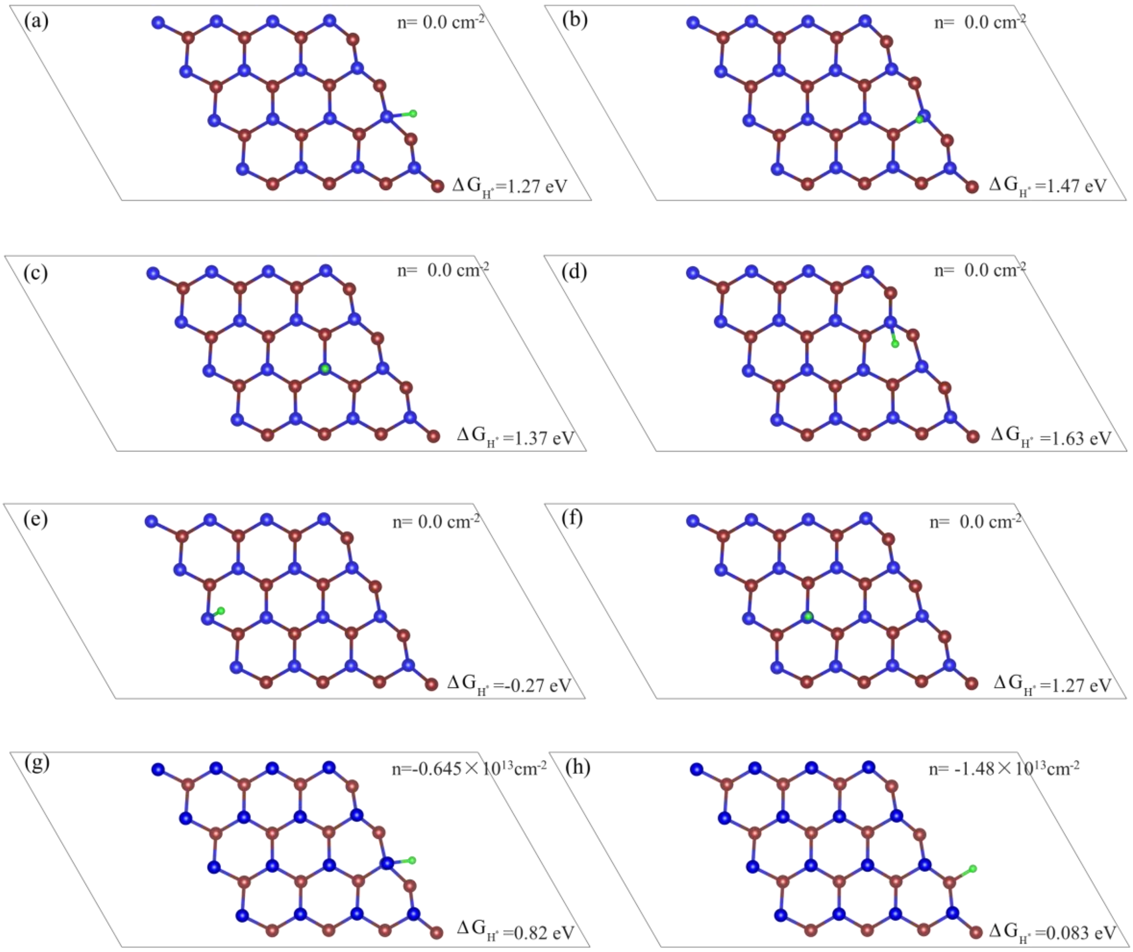

Figure S7. The effect of edge defect on the Gibbs free energy: (a)-(f) The optimized model with different adsorption sites. The effect of charge carrier concentration (per unit area) on the Gibbs free energy of the model (g), (h) n= -0.645×10^{13} cm^{-2}, (h) n= -1.48×10^{13} cm^{-2}.

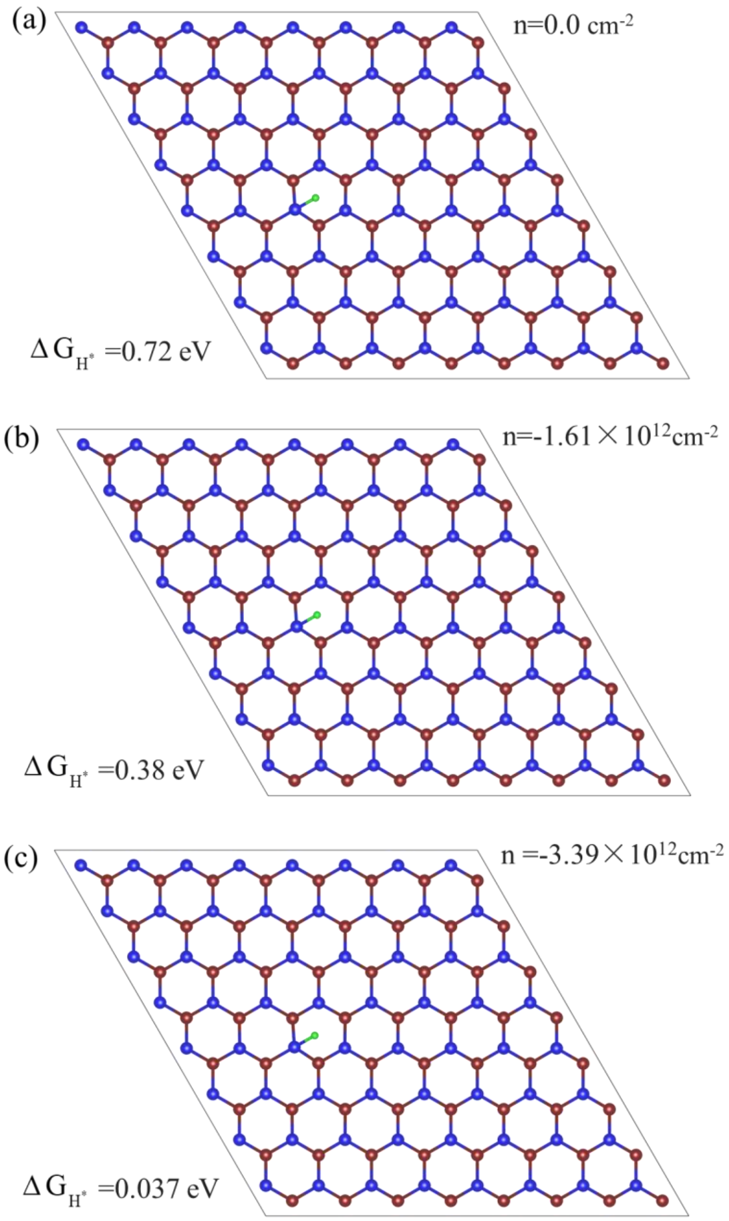

Figure S8. The effect of charge carrier concentration (per unit area) on the Gibbs free energy, based on a larger supercell with 8×8×1 unit cells (193 atoms). (a) n=0.0 cm^{-2}, (b) n= -1.61×10^{12} cm^{-2}, (c) n= -3.39×10^{12} cm^{-2}.
